# Supplementary material for: Unsupervised inference approach to facial attractiveness
Source: PeerJ. 2020 Oct 28;8:e10210. doi: 10.7717/peerj.10210 (PMC7602690; doi:10.7717/peerj.10210)
Supplement: Supplemental Information 2 — Includes a theoretical introduction to the Maximum Entropy method and a discussion regarding the MaxEnt inference in the presence of constraints among the vector coordinates. [file peerj-08-10210-s002.pdf]

Unsupervised inference approach to facial attractiveness SUPPLEMENTARY INFORMATION

Miguel Ibáñez-Berganza[1,*], Ambra Amico[2], Gian Luca Lancia[1],
Federico Maggiore[1], Bernardo Monechi[3], Vittorio Loreto[1,3,4]
[1] Sapienza University of Rome, Physics Department, Piazzale Aldo Moro 2, 00185 Rome, Italy.
[2] ETH Zurich, Chair of Systems Design, WEV G 212 Weinbergstrasse 56/58, 8092 Zurich.
[3] Sony Computer Science Laboratories, Paris, 6, rue Amyot, 75005, Paris, France.
[4] Complexity Science Hub, Josefstädter Strasse 39, A 1080 Vienna, Austria.
[*]miguel.ibanezberganza@gmail.com

Contents

0.1 Introduction to the Maximum Entropy principle: Correlations vs effective interactions 1
0.2 Maximum Entropy inference from pairwise correlations with a priori constraints . 3
0.3 Constraints in the database of facial modifications 5
0.4 Correlation vs interaction matrices 7
0.5 Longitudinal and Torsion interaction strengths 7
0.6 Dependence of J on inter-landmark distances and angles 8
0.7 The Harmonic inference in the limit $C^{(\mathrm{xx})}, C^{(\mathrm{yy})} \gg C^{(\mathrm{xy})}$ 9
0.8 Two ways of inferring with constraints in the database of facial modifications . . . 10
0.9 Average proportions and pairwise correlations 12
0.10 Harmonic interactions and elastic constants 14
0.11 Cognitive origin of non-linear correlations 14
0.12 Generality of the unsupervised inference models 15
0.13 Learning in the non-linear MaxEnt model. 15
0.14 Learning the database with the Gaussian Restricted Boltzmann Machine 16
0.15 Classification with the Random Forest algorithm 18
0.16 Detailed comparison among several classification methods 18
0.17 Inter- and intra-subject correlations and errors. 19
0.18 Description of the E1 experiment 20

1 Bibliography **20**

0.1 Introduction to the Maximum Entropy principle: Correlations vs effective interactions

Consider an n-dimensional space of vectors, $\mathbf{x} = (x_i)_{i=1}^n \in \chi$, along with a set of K observables, $O_k : \chi \to \mathbb{R}$, $k = 1, \ldots, K$. The *maximum entropy* approach [1, 2, 3, 4] provides the *most probable* probability distribution $P(\mathbf{x}|\boldsymbol{\lambda})$, $\mathbf{x} \in \chi$, which is consistent with a fixed value of the operators, in the sense that their average according to P, $\langle O_k \rangle_P$ is constraint to assume a fixed value,

$$\langle O_k \rangle_P = o_k \tag{0.1}$$

(where $\langle O_k \rangle_P = \int \mathrm{d}\mathbf{x}\, O_k(\mathbf{x}) P(\mathbf{x}|\boldsymbol{\lambda})$). In other words the *maximum entropy* probability distribution P_{me} is the one exhibiting highest entropy (i.e., the most random, or less structured distribution) subject to the constraint (0.1), and to no other constraint. It assumes the form:

$$P_{\mathrm{me}}(\mathbf{x}) = \frac{1}{Z(\boldsymbol{\lambda})} \exp\left[\sum_{k=1}^{K} \lambda_k O_k(\mathbf{x})\right] \tag{0.2}$$

$Z(\boldsymbol{\lambda})$ being a normalizing constant. The maximum entropy probability distribution is, hence, formally identical to a Maxwell-Boltzmann distribution in the canonical ensemble at temperature

$= 1$, with effective Hamiltonian $\mathcal{H} = -\sum_k \lambda_k O_k$. It is important to remark that no assumption at all has been done about thermal equilibrium, ergodicity, nor about the existence of an effective interaction in energy units: the Maxwell-Boltzmann form is a consequence of the maximum entropy assumption –reflecting, rather, *absence* of hypothesis– of a probability distribution subject to constraints on the average of some operators. The values of the Lagrange multipliers λ's in (0.2) are such that the constraints in (0.1) are satisfied.

In the context of unsupervised statistical inference, one infers from a finite number M of experimental measures of the observables O_k, to which correspond the values $o_k^{(m)}$, $m = 1, \dots, M$. The maximum entropy distribution provides a generative probabilistic model for the data, that is aimed to be a faithful representation of the experimental dataset and, at the same time, a *generalisation* of the dataset, not too dependent on the specific realisation of the database that is being inferred. For this reason, P is chosen to reproduce the experimental value of *a limited* set of observables, depending on the dataset. Ideally, a faithful and general model should be consistent with the minimum set of experimental averages that allow to reproduce some essential database properties and, at the same time, that may be significantly inferred given the database finiteness. Once the observables have been selected, a possible choice for *their value* o_k (determining the value of the parameters λ_k) in 0.1 is the experimental average, $\langle O_k \rangle = (1/M) \sum_{m=1}^{M} o_k^{(m)}$. This choice $o_k = \langle O_k \rangle$ is equivalent to the Maximum Likelihood prescription of the whole experimental database:

$$\{\lambda_k^*\}_k = \arg \max_{\{\lambda_k\}_k} \sum_{m=1}^{M} \ln P(\mathbf{x}^{(m)} | \boldsymbol{\lambda}) \tag{0.3}$$

where $\mathbf{x}^{(m)}$ is the m-th experimental configuration, and $o_k^{(m)} = O_k(\mathbf{x}^{(m)})$. The parameters λ are called effective interactions, in the language of statistical physics. In the case that the observables to be reproduced by P are the data correlations of order $n \le p$ (where the correlations of order n are defined as $C^{(n)}_{i_1,\cdots,i_n} = \langle x_{i_1} \cdots x_{i_n} \rangle)$, the effective interactions assume the form of n-th order tensors $J^{(n)}$ coupling n-plets of vector coordinates, with $n = 1, \dots, p$.

A self-consistency criterion for the choice of the sufficient statistics O_k is that of calculating different nontrivial observables according to P (different from the sufficient statistics, i.e., observables that P is not required to reproduce by construction, and that cannot be expressed in terms of the sufficient statistics), and comparing them with their experimental counterparts. In particular, a criterion is that of choosing the $n \le p$-th order correlations as sufficient statistics, with p being the minimum value such that the $p+1$-th order experimental correlations are satisfactorily reproduced by $P_{\rm me}$ (i.e., $\langle x_{i_1} \cdots x_{i_{p+1}} \rangle \simeq \langle x_{i_1} \cdots x_{i_{p+1}} \rangle_P$), and such that all the parameters corresponding to such sufficient statistics may be significantly inferred from the data.

Correlations and effective interactions. The effective interaction tensors $J^{(n)}$ may admit, in certain circumstances, an interpretation regarding the mutual effective influence among variables, beyond the statistical correlation among them (whose experimental value is $C^{(n)}$). Correlations and effective interactions are actually different. Focusing for simplicity in $p = 2$, the pairwise correlations are but the statistical consequence of the effective interactions among couples of landmarks causing them. This is the case in the direct problem (the calculation of $C^{(2)}$ from $J^{(2)}$): in this case, it may happen that the matrix $J^{(2)}$ is sparser than $C^{(2)}$: there are couples of variables not influencing each other that, nevertheless, result statistically correlated. In the direct problem, the Maximum Entropy method may allow for a discrimination of the spurious correlations of couples of components that are correlated although they do not influence each other (but are, instead, commonly and mutually influenced by other components). This a frequent phenomenon in biological data [5, 6], with an obvious interpretation in statistical physical terms: in the general case, the mutual influence among a sparse set of couples of bodies propagates statistically, leading to *emergent, collective phenomena*. A paradigmatic and extreme case of this general phenomenology is critical behaviour [7], in which microscopic interactions lead to macroscopic correlations: long-range and high-order body correlations originate from short-range, sparse and pairwise interactions [5]).

Probably the simplest illustration of the emergence of spurious statistical correlations is that of three variables (x_1, x_2, x_3 in fig. 0.1, of which only two of them are strongly interacting (in the figure $J_{12} = J_{13} = 2$), while the second and third are moderately interacting (or even negatively interacting, as in the figure: $J_{23} = -1$). Such an information is not accessible from the emerging

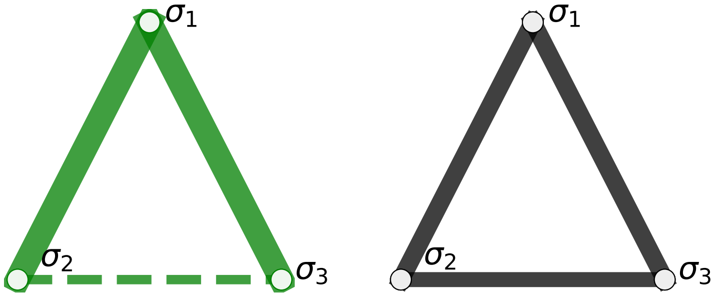

Figure 0.1: Effective interactions (left) versus the emerging statistical correlations (right) among three binary variables, $\sigma_i = \pm 1$, $i = 1, 2, 3$. The effective interactions are given by the symmetric matrix $J_{12} = J_{13} = 2$, $J_{23} = -3/4$ (i.e., 2,3 are negatively coupled). The correlations $\langle \sigma_i \sigma_j \rangle$ are given by their expectation value according to the Maxwell-Boltzmann probability distribution corresponding to a pairwise interaction given by matrix J: $\langle \sigma_i \sigma_j \rangle = \sum_{\boldsymbol{\sigma}} \sigma_i \sigma_j P(\boldsymbol{\sigma})$, with $P(\boldsymbol{\sigma}) = \exp(+\sum_{i<j} \sigma_i \sigma_j J_{ij})/Z$ where $Z = \sum_{\boldsymbol{\sigma}} \exp(+\sum_{i<j} \sigma_i \sigma_j J_{ij})$. The line width is proportional to the absolute value $|A_{ij}|$ of the corresponding matrix element. The dashed line in the J triangle indicates that J_{23} is negative (i.e., there is a tendency of 2 to decrease when 3 increases and vice-versa). Such tendency is, however, not reflected in the correlation matrix, which presents all positive elements.

correlations $\langle x_i x_j \rangle_{P(\cdot|J)}$ (in the direct problem), revealing a strong, positive correlation among all the variables. Conversely, in the inverse problem (i.e., when an empirical correlation matrix is given, resulting from an average of a sufficiently high number of measures), the Maximum Entropy method may provide not only a generative model, $P(\cdot|J_*^{(n)})$, but also the most probable interaction matrices $J_*^{(n)}$ suggesting that, indeed, the correlation among 2 and 3 is (most likely, given the data and the sufficient statistics, and within the Maximum Likelihood hypothesis) a statistical consequence of the mutual influences of $1, 2$ and of $1, 3$. This information is not unambiguously elicited from the data, but the result of an inference procedure: the most probable guess given the inference model and the ambiguity induced by the data finiteness.

0.2 Maximum Entropy inference from pairwise correlations with a priori constraints

In this section we solve the problem of the Maximum Entropy (MaxEnt) inference from pairwise correlations (i.e., $p = 2$), in the presence of linear constraints involving the coordinates.

In the absence of constraints, the Maximum Likelihood solution to the problem, equation (0.3) is analytic and straightforward. Suppose that one infers from a database composed by S experimental realisations $\{\mathbf{x}^{(s)}\}_{s=1}^{S}$ of a real, D-dimensional vector, $\mathbf{x} = (x_i)_{i=1}^{D} \in \mathbb{R}^D$. The sufficient statistics to infer from is by hypothesis the correlation matrix (supposing null-average vectors): $C_{ij} = \langle x_i x_j \rangle$ where $\langle \cdot \rangle$ represents, as before, the experimental average: a symmetric, positive definite matrix. The MaxEnt probability distribution is, consequently, the multi-variate normal distribution:

$$P(\mathbf{x}|J) = \left[\frac{\det J}{(2\pi)^n} \right]^{1/2} \exp[-\frac{1}{2} \mathbf{x}^\dagger J \mathbf{x}]. \tag{0.4}$$

The Maximum Likelihood solution for the matrix J, J^*, is that satisfying that the theoretical pairwise correlations $\langle x_i x_j \rangle_P = {J^{-1}}_{ij}$ coincide with the experimental correlations C_{ij}. This is satisfied whenever $J^* = C^{-1}$.

We now consider the presence of linear constraints involving the coordinates, x_i. Each linear constraint may be expressed in the form $\boldsymbol{a}_j^\dagger \mathbf{x} = c_j$, being $\boldsymbol{a}_j$ a real D-dimensional vector and c_j a real constant, for the j-th constraint. If all the vectors in the database $\{\mathbf{x}^{(s)}\}_{s=1}^{S}$, are subject to the constraints, each constraint induces a null mode (a zero eigenvalue) in the experimental covariance matrix. In this case, the Maximum Likelihood solution to the problem, i.e., the probability

distribution $P(\cdot|J)$ such that $\langle x_i x_j \rangle_P = C_{ij}$ cannot simply be $J^* = C^{-1}$, since matrix C actually exhibits a vanishing determinant.

We will see that Maximum Likelihood solution in this case is $J^* = C^{-1}$, where the -1 exponent means the pseudo-inverse operation, a generalisation of the matrix inverse operation in which the null eigenvalues are avoided. We define the pseudo-inverse of the real, square matrix A as:

$$A^{-1}{}_{ij} = \sum_{k|\epsilon_k \neq 0} \epsilon_k^{-1} \eta_i^{(k)} \eta_j^{(k)} \tag{0.5}$$

where ϵ_k, $\eta_j^{(k)}$ are the k-th eigenvalue and the j-th component of the k-th eigenvector of A, respectively.

We first consider the solution of the direct problem, $\langle x_i x_j \rangle_P$ from J, in a situation in which the interaction matrix J is such that $\text{rank}(J) = r < D$. In other words, J exhibits $D - r$ null eigenvalues. Suppose that the eigenvalues λ_j of J are ordered in decreasing order, so that $\lambda_j = 0$ for $j = r+1, \ldots, D$. In this case, the probability distribution $P(\mathbf{x})$ in equation 0.4 is, trivially, constantly zero since the determinant of matrix J vanishes. However, we can define a real function in the space of the D-dimensional variables $\mathbf{x}$:

$$\tilde{P}(\mathbf{x}) = \frac{1}{\tilde{Z}} \exp[-\mathbf{x}^\dagger J \mathbf{x}] \tag{0.6}$$

where $\tilde{Z}$ is a normalising factor involving the non-zero eigenvalues of J only:

$$\tilde{Z} = \left[\frac{\tilde{\det} J}{(2\pi)^r} \right]^{1/2} \qquad \tilde{\det} J = \prod_{k=1}^{r} \lambda_k \tag{0.7}$$

The function $\tilde{P}$ may be considered as a normalised probability distribution, but only over the r-dimensional subspace of $\mathbb{R}^D$ expanded by the first r eigenvectors of J: $\mathbb{S}_+ = \text{span}\{\mathbf{e}^{(k)}\}_{k=1}^r$ with $1 \leq k \leq r$. In other words, $\tilde{P}$ is a probability distribution on the subspace of $\mathbb{R}^D$, $\mathbb{S}_+$, defined by the vectors that are already subject to the constraints, for any value c_j's of the constants associated to the constraints.

One can easily define a proper, normalised probability distribution P defined in $\mathbb{R}^D$, by regularising the null modes associated to the constraints:

$$P(\mathbf{x}) = \tilde{P}(\mathbf{x}) \prod_{j=r+1}^{D} \delta(x'_j - \mathsf{c}_j) \tag{0.8}$$

where the $D-r$-dimensional vector $(x'_{r+1}, \ldots, x'_D)$ is a vector of the projection of $\mathbf{x}$ in a basis of vectors expanding the space of the constraints (as the vectors $\mathbf{a}_j$ defining the constraints, before, $x'_{j+r} = \mathbf{a}_j^\dagger \mathbf{x}$). On the other hand, we define the r-dimensional vector $\mathbf{x}' = (x'_1, \ldots, x'_r)$ as the projection of $\mathbf{x}$ over the first r eigenvectors of J (associated to a non-null eigenvalue): $\mathbf{x}' = E\mathbf{x}$, where E is the $r \times D$ matrix defined as the row-disposed eigenvectors, $E_{ij} = e_j^{(i)}$[1].

To each of the null eigenvalues corresponding to a constraint $\mathbf{a} \cdot \mathbf{x} = c$, is associated an *invariance* of $\tilde{P}$ with respect to the linear operator $G(c)$ that changes the value of the constraint, i.e., such that $\mathbf{a} \cdot (G(c)\mathbf{x}) = c$:

$$\tilde{P}(\mathbf{x}|J) = \tilde{P}(G(c)\mathbf{x}|J). \tag{0.9}$$

Indeed, G acts on the subspace $\mathbb{S}_0$ only, while it is the identity in the subspace $\mathbb{S}_+$ (where $\mathbb{S}_0$ is defined as the complement of $\mathbb{S}_+$, i.e., $\mathbb{R}^D = \mathbb{S}_+ \times \mathbb{S}_0$). In the physical language, each eigenvector corresponding to a constraint is called a *null mode*, and represents a *symmetry* reflected in the invariance of the function $\tilde{P}$ with respect to the symmetry. The function P, in its turn, represents vectors for which the symmetry is broken, as the value of the constraint has been fixed. For example, if the vectors are constrained to have a constant sum of its components, $\sum_{i=1}^D x_i = c$, the corresponding eigenvector, or null mode $\mathbf{e}$, has all its components equal to $e_i = D^{-1/2}$. Consequently, the function $\tilde{P}$ is invariant under scale transformations.

We are now interested in the calculation of a general n-order cumulant $\langle\langle x_{s_1} \cdots x_{s_n} \rangle\rangle_P$ according to the distribution P in (0.8), with $s_j = 1, \ldots, D$. As it can be seen immediately, the n-th order

[1]We make notice that $EE^\dagger = \mathbb{I}_r$ but $E^\dagger E \neq \mathbb{I}_D$ (where $\mathbb{I}_d$ is the identity matrix in d dimensions).

cumulant is related to the n-th order derivative of the generating function through the standard cumulant expansion equation:

$$\langle\langle x_{s_1} x_{s_2} ... x_{s_n} \rangle\rangle_P = \left. \frac{\partial^n \ln \tilde{Z}[\mathbf{h}']}{\partial h'_{s_1} \partial h'_{s_2} ... \partial h'_{s_n}} \right|_{\mathbf{h}=\mathbf{0}} \tag{0.10}$$

where the generating function $\tilde{Z}[\mathbf{h}]$ has the form:

$$\tilde{Z}[\mathbf{h}] = \left[\prod_{k=1}^{r} \int_{-\infty}^{+\infty} dz'_k \right] e^{-\frac{1}{2}\mathbf{x}^\dagger J \mathbf{x} + \mathbf{h}^\dagger \mathbf{x}} \tag{0.11}$$

We notice that $\tilde{Z}[\mathbf{0}] = \tilde{Z}$. We would like an analytical expression for $\tilde{Z}[\mathbf{h}]$. Using the relations $\mathbf{x}' = E\mathbf{x}$ and $J = E^\dagger \Lambda E$, where Λ is the $r \times r$ diagonal matrix whose diagonal is $\lambda_1, \ldots, \lambda_r$, one obtains:

$$\tilde{Z}[\mathbf{h}] = \prod_{k=1}^{r} \int_{-\infty}^{+\infty} dx'_k \exp\left[-\frac{1}{2} x'^2_k \lambda_k + x'_k h'_k \right] \tag{0.12}$$

where $\mathbf{h}' = E\mathbf{h}$. Using Gaussian integration rules, one finds:

$$\tilde{Z}[\mathbf{h}] = \tilde{Z} e^{\frac{1}{2}\mathbf{h}^\dagger J^{-1} \mathbf{h}} \tag{0.13}$$

where J^{-1} is the pseudo-inverse of matrix J, ${J^{-1}}_{ij} = \sum_{k \le r} \lambda_k^{-1} e_i^{(k)} e_j^{(k)}$. This equation is the generalisation of the standard expression for the generating function of the multi-variate normal distribution, to the case in which matrix J is be non-invertible. As it is evident from the expression of $\tilde{Z}[\mathbf{h}]$ and from the cumulant expansion (0.10), and as it happens with the normal distribution, the only non-zero cumulant is the second-order cumulant $\langle\langle x_i x_j \rangle\rangle_P$, equal to the correlation $\langle x_i x_j \rangle_P$ in the case of null-averaged vectors. Its form is, from equation 0.10:

$$\langle x_i x_j \rangle_P = {J^{-1}}_{ij} \tag{0.14}$$

where J^{-1} is the pseudo-inverse of matrix J.

The theoretical correlation matrix whose elements are $\langle x_i x_j \rangle_P$ exhibits, as matrix J does, rank equal to r. Hence, the theoretical correlation matrix $M_{ij} = \langle x_i x_j \rangle$ as a function of J (i.e., the direct problem) is $M = J^{-1}$. Consequently, the J^* that is needed to satisfy $C_{ij} = \langle x_i x_j \rangle_P$ given an experimental correlation matrix C (i.e., the inverse problem) is $J^* = C^{-1}$, where the -1 power means the pseudo-inverse operation.

0.3 Constraints in the database of facial modifications

As we have explained in the main text, the facial modifications in the 2017 experiment are defined in terms of a set of 17 2D landmarks which are redundant in the sense that the positions of some of them may be deduced in terms of 10 coordinates only. The facial landmarks are, in particular, symmetric by construction, and hence the coordinates of the right-side landmark are determined given those of the left side. We have, hence, considered a subset of $n = 8$ landmarks only (see figure 0.2 Furthermore, the landmark coordinates $r_{\mathsf{c},i}$ (where $\mathsf{c} = \mathsf{x}, \mathsf{y}$) in the database $\mathcal{S}$ are still subject to 6 constraints: indeed, $2 \times n - 6 = 10$, the numer of degrees of freedom. For instance, the nose endpoint abscissa is constrained to lie in the center of the image, $\Delta_{\mathsf{x},9} = 0$, and the jaw landmark is defined to be at the same heigth of the mouth, $\Delta_{\mathsf{y},3} = \Delta_{\mathsf{y},7}$. We have intentionally kept such redundant information in the inferred training database. Indeed, the redundant information turns to be necessary for the correct *interpretation* of the inference parameters, as we will see in section 0.8.

The database $\mathcal{S} = \{\mathbf{\Delta}^{(s)}\}_{s=1}^{S}$ of facial displacements is, hence, highly constrained in the various facial coordinates. The following constraints hold, for all the vectors in the database:

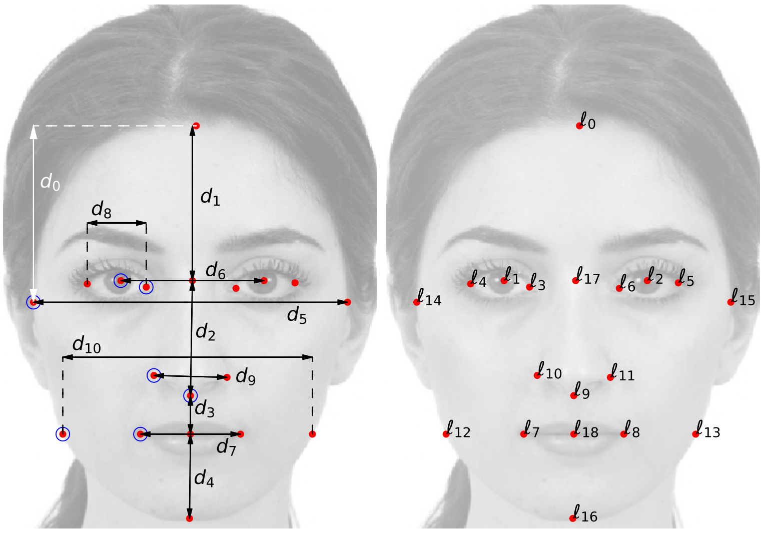

Figure 0.2: Left: definition of the face space in terms of inter-landmark distances $\mathbf{d}$. The landmark coordinates are, instead, the x, y 2D coordinates $\vec{r}_i$ of a subset of seven landmarks, marked with blue circles. Right: all the landmarks used for the facial deformation algorithm described in [8].

$$\Delta_{4,\mathsf{x}} = 0 \tag{0.15}$$
$$\Delta_{4,\mathsf{y}} - \Delta_{5,\mathsf{y}} = \text{constant} \tag{0.16}$$
$$\Delta_{7,\mathsf{y}} - \Delta_{3,\mathsf{y}} = 0 \tag{0.17}$$
$$\frac{\Delta_{0,\mathsf{y}} - \Delta_{1,\mathsf{y}}}{\Delta_{0,\mathsf{x}} - \Delta_{1,\mathsf{x}}} = \text{constant} \tag{0.18}$$
$$\frac{\Delta_{0,\mathsf{y}} - \Delta_{2,\mathsf{y}}}{\Delta_{0,\mathsf{x}} - \Delta_{2,\mathsf{x}}} = \text{constant} \tag{0.19}$$
$$\frac{\Delta_{1,\mathsf{x}} - \Delta_{0,\mathsf{x}}}{\Delta_{0,\mathsf{x}} - \Delta_{2,\mathsf{x}}} = \text{constant} \tag{0.20}$$

The last three constraints ensure that the eye aspect ratio remains unchanged with respect to the average facial vector $\mathbf{\Delta} = \mathbf{0}$ (otherwise, the image deformation algorithm corresponding to the landmark deformation $\mathbf{0} \to \mathbf{\Delta}$ could lead to an ellipse-like shaped eye). Indeed, the constants in the right-hand side of each equation correspond to the value that assumes the left-hand side quantity in the average facial vector.

Each of these constraints induces a null mode in one of the correlation matrices $C^{(\mathsf{xx})}$, $C^{(\mathsf{yy})}$, $C^{(\mathsf{xy})}$ (those involving only x's coordinates, in $C^{(\mathsf{xx})}$; those involving only y's, in $C^{(\mathsf{yy})}$; those involving a x and a y coordinate, in $C^{(\mathsf{xy})}$). As we have shown before, the inverse problem in this case is solved through the matrix pseudo-inverse operation. In these circumstances, the probability distribution $\mathcal{L}(\cdot|J, \mathbf{h})$ described in the main article refers to a probability distribution in the 10-dimensional sub-space of coordinates that are invariant under the symmetries associated to the constraints ($\tilde{P}$, in the notation of section 0.2). Strictly speaking, to become a proper probability distribution in the space of facial modification vectors $\mathbf{\Delta}$ it has to be regularised as in section 0.2:

$$P(\mathbf{\Delta}|J, \mathbf{h}) = \left(\prod_{\mu} \delta(\Delta'_{\mu} - \mathsf{c}_{\mu}) \right) \tilde{P}(\mathbf{\Delta}|J, \mathbf{h}) \tag{0.21}$$

where $\tilde{P}$ is the distribution that in the main article is called $\mathcal{L} = \exp(-H)/Z$, the product is over the $\mathbf{\Delta}$ components over the 6 eigenvectors of the global correlation matrix with a null eigenvalue, $\lambda_{\mu} = 0$, and $\mathbf{\Delta}' = E\mathbf{\Delta}$, with E being the matrix of column-eigenvectors of C (and of J).

0.4 Correlation vs interaction matrices

In the particular case of our database, the main source of spurious correlations is not collective behaviour but the presence of the *a priori* constraints among various landmark coordinates, which are imposed in the experimental construction of the face space vectors (see sec. 0.3 and [8] for a precise description of the constraints), and that play the role of the strong interaction $1, 2$ in fig. 0.1. The MaxEnt method *subtracts* the effect of such constraints and provides a sparser interaction matrix. Our MaxEnt inference scheme discounts the effect of constraints since we eliminate the matrix C eigenvectors corresponding to the constraints (through the pseudo-inverse operation C^{-1}), see sec 0.8 for an in-depth discussion.

In figure 0.3 we present a comparison among the matrices $C^{(\mathsf{xx})}$ and $J^{(\mathsf{xx})}$, $C^{(\mathsf{yy})}$ and $J^{(\mathsf{yy})}$, $C^{(\mathsf{xy})}$ and $J^{(\mathsf{xy})}$. As a general observation, the effective matrices are sparser than the correlation matrices, as expected. In particular, while both ${C^{(\mathsf{xx})}}_{6,3}$ and ${C^{(\mathsf{xx})}}_{7,3}$ are statistically significant, only $J_{7,3}$ is (the effective interaction attributes the $6-3$ correlation to the $\Delta_{7,\mathsf{x}} - \Delta_{3,\mathsf{x}} = 0$ constraint). The same happens, for instance with ${C^{(\mathsf{yy})}}_{1,3}$ and ${C^{(\mathsf{yy})}}_{0,3}$, statistically significant, while only $J_{0,3}$ is (the $1-3$ correlation is attributed to the $0-1$ constraint).

We conclude that the effective interaction coupling matrix J provides information beyond the experimental correlations, since it disambiguates the correlations propagated by the constraints, attributing them to the effect of a reduced set of couplings. In section 0.8 we illustrate the fact that an alternative method of avoiding the constraints, consisting in fitting a dataset in which the redundant variables are eliminated (instead of keeping them and avoiding the influence of the constraint-eigenvectors), may lead to J matrices whose interpretation is misleading.

0.5 Longitudinal and Torsion interaction strengths

The $n \times n$ vertical, horizontal and oblique correlation matrices are defined as the corresponding correlations among landmark fluctuations: ${C^{(\mathsf{xx})}}_{ij} = \langle \Delta_{i,\mathsf{x}} \Delta_{j,\mathsf{y}} \rangle$, and the same for $C^{(\mathsf{xy})}$, $C^{(\mathsf{yy})}$. The whole $2n \times 2n$ correlation matrix C is defined as $C_{\mu\nu} = \langle \Delta_\mu \Delta_\nu \rangle$, where the $2n$ Greek indices $\mu = i, \mathsf{c}_i$ denote the $\mathsf{c}_i = \mathsf{x}, \mathsf{y}$ coordinates of the i-th landmark. We define analogously the vertical, horizontal and oblique interaction matrices. The relation among these matrices is given by:

$$C = \begin{pmatrix} C^{(\mathsf{xx})} & C^{(\mathsf{xy})} \\ C^{(\mathsf{xy})\dagger} & C^{(\mathsf{yy})} \end{pmatrix}, \qquad J = \begin{pmatrix} J^{(\mathsf{xx})} & J^{(\mathsf{xy})} \\ J^{(\mathsf{xy})\dagger} & J^{(\mathsf{yy})} \end{pmatrix}, \qquad C = J^{-1} \tag{0.22}$$

where the -1 power means the pseudo-inverse operation.

In their turn, the longitudinal and torsion interaction matrices, $J^{\parallel}$, $J^{\perp}$, correspond to the displacements along, and normal to, the segment joining the landmarks i and j, called $\hat{e}_{ij} = \langle \vec{r}_{ij} \rangle / r_{ij}$, where $\vec{r_{ij}} = \vec{r}_j - \vec{r}_i$ and $r_{ij} = |\langle \vec{r}_{ij} \rangle|$. These are defined so that the matrix elements $J^{\parallel}_{ij}$, $J^{\perp}_{ij}$ are the ${J^{(\mathsf{xx})}}_{ij}$ and ${J^{(\mathsf{yy})}}_{ij}$ couplings, but in a ($ij-$dependent) rotated basis such that the x-axis coincides with the i, j inter-landmark segment versor, $\hat{e}_{ij}$. Henceforth, the $J^{\parallel}$ and $J^{\perp}$ matrices are not obtained by a rotation of the original matrices $J^{(\mathsf{xx})}$ and $J^{(\mathsf{yy})}$. Instead, each $J^{\parallel}_{ij}$ element results from a whole inference procedure in a different basis depending on the couple ij. In particular, $J^{\parallel}_{ij} = {J^{(\mathsf{xx})}}_{ij}(\hat{e}_{ij})$, where $J^{(\mathsf{xx})}(\hat{e}_{ij})$ is the inferred matrix obtained from the pseudo-inverse of matrix $C(\hat{e}_{ij})$ in a coordinate system in which the x axis coincides with the ij-segment (in other words, $C(\hat{e}_{ij}) = \mathcal{R}^{\dagger}_{ij} C \mathcal{R}_{ij}$, where $\mathcal{R}_{ij}$ is the 2D rotation matrix by the angle $-\alpha_{ij}$, and the matrix product is over the x and y blocks of matrix C).

We remark that there is less information in $J^{\parallel}_{ij}$, $J^{\perp}_{ij}$ (for all i, j) than in the whole effective interaction matrix J (since the matrix whose matrix elements are ${J^{(\mathsf{xy})}}_{ij}(\hat{e}_{ij})$ is not $J^{\parallel}$, nor $J^{\perp}$).

We now provide a clearer interpretation of the longitudinal and torsion effective interaction matrices. $J^{\parallel}_{ij}$, $J^{\perp}_{ij}$ capture the relative relevance of the fluctuations around the average distance $\langle r_{ij} \rangle$, and of angle fluctuations around α_{ij}, respectively. Large values of $J^{\parallel}_{ij}$ imply that the distance among i and j in the direction of its average axis is highly "locked", i.e., it tends to exhibit small fluctuations, from sample to sample, around its most probable value. For instance (see figure 4 in the main text), a small fluctuation $\delta^{\parallel}_{4,7} = |(\vec{\Delta}_4 - \vec{\Delta}_7) \cdot \hat{e}_{4,7}|$ of the $4, 7$-segment distance with respect to the average face $\mathbf{\Delta} = \mathbf{0}$ implies a large energy increment, $J^{\parallel}_{4,7} {\delta^{\parallel}_{4,7}}^2$ and, consequently, a large decrement of the probability density $\mathcal{L}$, proportional to $\exp(-J^{\parallel}_{4,7} {\delta^{\parallel}_{4,7}}^2)$. Conversely, a

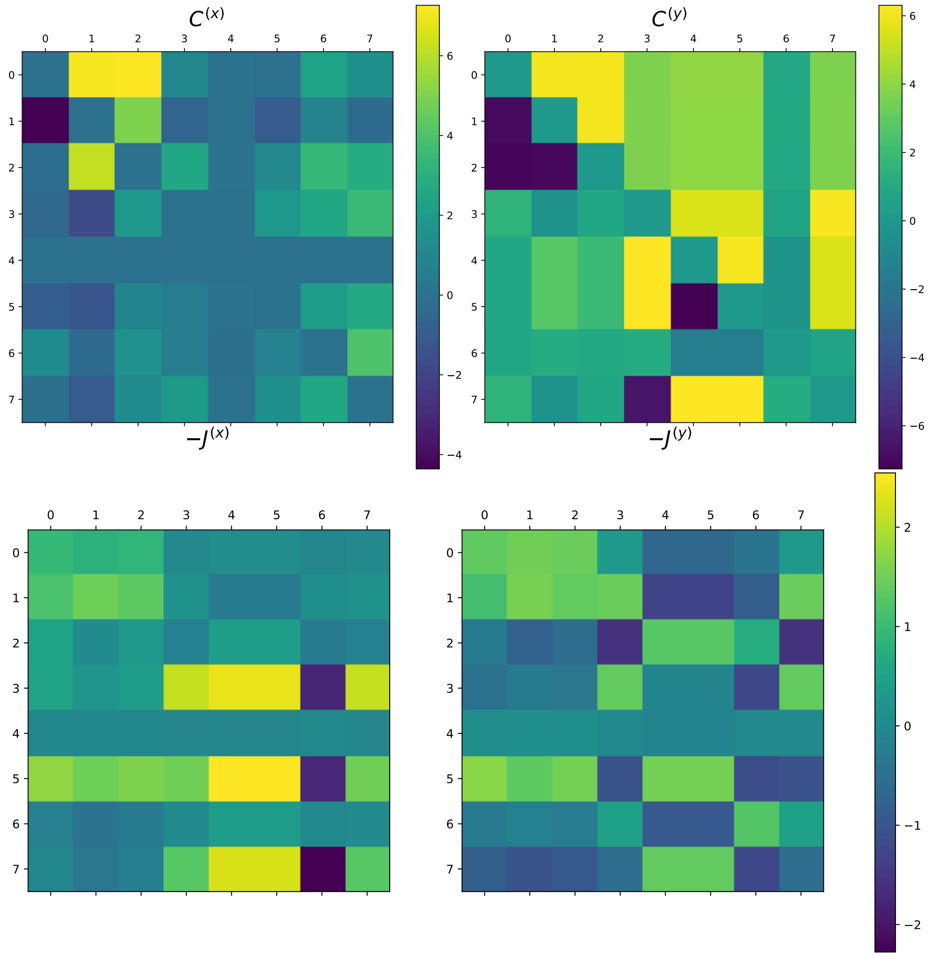

Figure 0.3: Comparison between matrices C and J. Top left: the $C^{(\mathrm{xx})}$ box of matrix C (upper right triangle) versus the $J^{(\mathrm{xx})}$ box of matrix $-J$. Top right: idem, but for $C^{(\mathrm{yy})}$ and $J^{(\mathrm{yy})}$. Bottom left: $C^{(\mathrm{xy})}$. Bottom right: $J^{(\mathrm{xy})}$. Mind that the J matrix is such that negative matrix elements represent ferromagnetic couplings, or affine interactions. As a general trend, matrix J is sparser than matrix C, as expected. The matrices C and J exhibit similar matrix elements, except in couples of coordinates involved in the same constraint. In the top row, the diagonal has been set to zero.

fluctuation of the $6,7$ segment distance $\delta^{\parallel}_{6,7}$ will give rise to a small or non-significant decrement of the probability of the resulting facial vector since the longitudinal coupling constant $J^{\parallel}_{6,7}$ is small, a fact that highlights the prominent importance of the inter-landmark distance $r_{4,7}$ over $r_{6,7}$ in the process of facial discrimination. In the same way, fluctuations in the transversal components of both segments, $\delta^{\perp}_{4,7}$ and $\delta^{\perp}_{6,7}$ (and consequent fluctuations of the inter-landmark segment angles around $\alpha_{4,7}$ and $\alpha_{6,7}$), have a strong impact in their probability of being sculpted (i.e., in their perceived attractiveness), since both torsion coupling constants $J^{\perp}_{4,7}$ and $J^{\perp}_{6,7}$ are large in absolute value.

0.6 Dependence of J on inter-landmark distances and angles

A different, interesting aspect of the matrix of effective interactions J is the dependence of the interaction strengths among landmarks i, j as a function of their average distance $\langle r_{ij} \rangle$ and average segment angle α_{ij}. We stress that $\langle r_{ij} \rangle$ and α_{ij} are meta-parameters in the sense that that they are not codified in the database $\mathcal{S}$ and, hence, are not inferred (the facial vectors $\mathbf{\Delta}$ are actually fluctuations around the average single-landmark positions). From a cognitive point of view, one would expect that the interaction strengths $|J^{(\mathrm{xx})}{}_{ij}|$, $|J^{(\mathrm{yy})}{}_{ij}|$, $|J^{(\mathrm{xy})}{}_{ij}|$ among couples of nearby landmarks should tend to be stronger for smaller values of r_{ij} or, at most, that they do not present

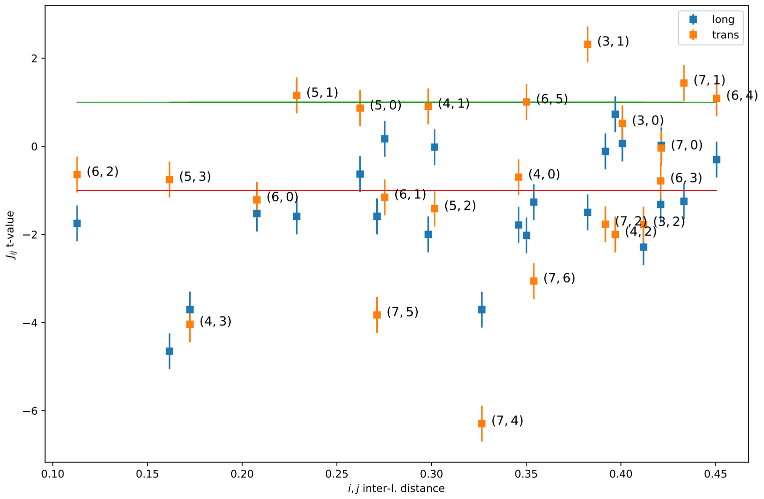

Figure 0.4: The t-value corresponding to the matrix elements $J^{\|}_{ij}$, $J^{\perp}_{ij}$ versus the inter-landmark average distance $\langle r_{ij} \rangle$.

an increasing trend (which would mean that farther away landmarks influence each other more than closer landmarks). In its turn, if the ij coupling absolute value decreases with α_{ij}, this would indicate the prominence of horizontal over vertical inter-landmark segments, and vice versa.

The data does not allow for sharp conclusions at these regards. However, and although the absolute value of the J matrix elements do not show a clear trend with r_{ij} nor with α_{ij}, some interesting information can be retrieved from such analysis. Indeed, a moderate decreasing trend is observed in $|J^{\|}_{ij}|$ vs. r_{ij}, signifying that nearer landmarks tend to influence each other more than farther away landmarks, but *only along the inter-ij landmark segment*, in the sense that only the longitudinal coupling presents such trend. Interestingly, the trend is lost when the x, y, xy components of J are plotted vs. r_{ij}. The absence of a clear trend with α_{ij} indicates lack of prominent importance of horizontal versus vertical inter-landmark segments.

We show in figures 0.4,0.5 the quantities $J^{\|}_{ij}$, $J^{\perp}_{ij}$ versus $\langle r_{ij} \rangle$ and α_{ij}, respectively (see the main article). Although no clear trend is observed, it is apparent a moderate decreasing trend of $|J^{\|}_{ij}|$ versus $\langle r_{ij} \rangle$, as referred in the main article, and a slight decreasing trend of $|J^{\perp}_{ij}|$ versus α_{ij}.

We notice that, in the notation of the article, negative values of J indicate the tendency to positive correlations (a ferromagnetic interaction, in the statistical-physical language).

0.7 The Harmonic inference in the limit $C^{(\mathsf{xx})}, C^{(\mathsf{yy})} \gg C^{(\mathsf{xy})}$

It can be shown that the solution of the inverse problem, at first order in the limit $C^{(\mathsf{xy})} \ll C^{(\mathsf{xx})}, C^{(\mathsf{yy})}$), is:

$$J^{(\mathsf{xx})} = {C^{(\mathsf{xx})}}^{-1} \tag{0.23a}$$

$$J^{(\mathsf{yy})} = {C^{(\mathsf{yy})}}^{-1} \tag{0.23b}$$

$$J^{(\mathsf{xy})} = -J^{(\mathsf{xx})} C^{(\mathsf{xy})} J^{(\mathsf{yy})} \tag{0.23c}$$

and that, in this limit, it is:

$$Z = \frac{(2\pi)^n}{(\det J^{(\mathsf{xx})}\ \det J^{(\mathsf{yy})}\ \det J^{(\mathsf{xy})})^{1/2}} \tag{0.24}$$

This can be shown by Gaussian integration, or approximating the inverse of the matrix

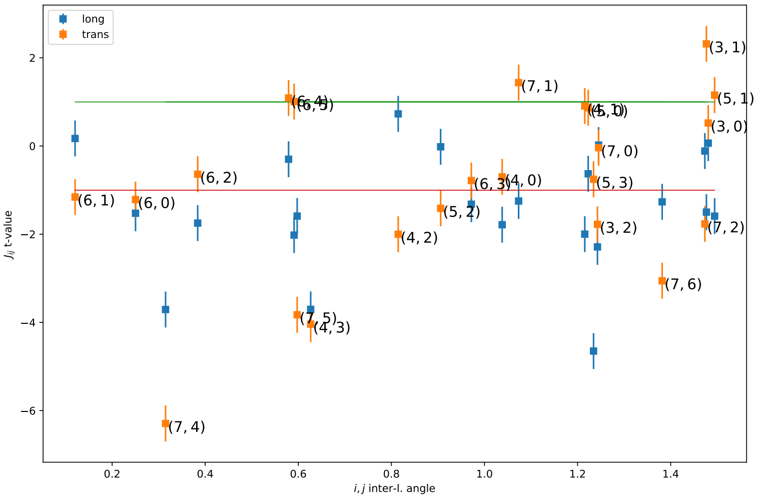

Figure 0.5: The t-value corresponding to the matrix elements $J_{ij}^{\|}$, $J_{ij}^{\perp}$ versus the angle subtended by $\hat{e}_{(ij)}$ with the x-axis, α_{ij}.

$$J = \begin{pmatrix} J^{(\mathrm{xx})} & J^{(\mathrm{xy})} \\ J^{(\mathrm{xy})\dagger} & J^{(\mathrm{yy})} \end{pmatrix} \tag{0.25}$$

by using the first-order (in A) matrix expansion: $[B(1{+}A)]^{-1} \simeq (1{-}A)B^{-1}$, with $B = \begin{pmatrix} J^{(\mathrm{xx})} & 0 \\ 0 & J^{(\mathrm{yy})} \end{pmatrix}$.

Indeed, the experimental matrices $C^{(\mathrm{xx})}$, $C^{(\mathrm{xx})}$ are larger than $C^{(\mathrm{xy})}$. The approximated solution, equation 0.23 is, consequently, a rather good approximation. In figure 0.6 we show this by comparing the exact $J^{(\mathrm{xx})}$, $J^{(\mathrm{yy})}$, $J^{(\mathrm{xy})}$ as different blocks of $J = C^{-1}$, versus the ones resulting from equation (0.23).

The relative influence of oblique correlations may be also assessed by defining a simpler model, that we will call the *null*-xy *model*, consisting in neglecting oblique interaction terms (taking $J^{(\mathrm{xy})} = 0$). An even simpler model, that we will call *dot model*, consists in neglecting oblique interactions and supposing that the couplings $J^{(\mathrm{xx})}$ and $J^{(\mathrm{yy})}$ are equal:

$$H_{\mathrm{dot}} = \frac{1}{2} \sum_{i,j} J_{ij}^{(\mathrm{dot})} \, \vec{\Delta}_i \cdot \vec{\Delta}_j \tag{0.26}$$

In this case the probability distribution is simply:

$$P_{\mathrm{dot}}(\mathbf{\Delta}_{\mathrm{x}}, \mathbf{\Delta}_{\mathrm{y}} | J^{(\mathrm{dot})}) = \frac{(2\pi)^n}{\det J^{(\mathrm{dot})}} \exp\left(-H_{(\mathrm{dot})}[\mathbf{\Delta}_{\mathrm{x}}, \mathbf{\Delta}_{\mathrm{y}}]\right) \tag{0.27}$$

where $J^{(\mathrm{dot})}$ is the inverse matrix of $C_{ij}^{(\mathrm{dot})} = \langle \vec{\Delta}_i \cdot \vec{\Delta}_j \rangle$.

We have assessed the efficiency of the dot and null-xy models by evaluating their efficiency in the classification task. As we show in section 0.16, neglecting the oblique correlations (in the null-xy model) and the anisotropy of vertical/horizontal correlations (in the dot model) leads to a poorer performance. This provides a quantitative assessment of the relative influence of these terms. We conclude that *the influence of oblique correlations is crucial, and not negligible, in the facial perception process.*

0.8 Two ways of inferring with constraints in the database of facial modifications

In section 0.2, we have exposed a method of MaxEnt inference (from pairwise interactions) from a database exhibiting linear constraints. Within this method, all the D components of the vectors are

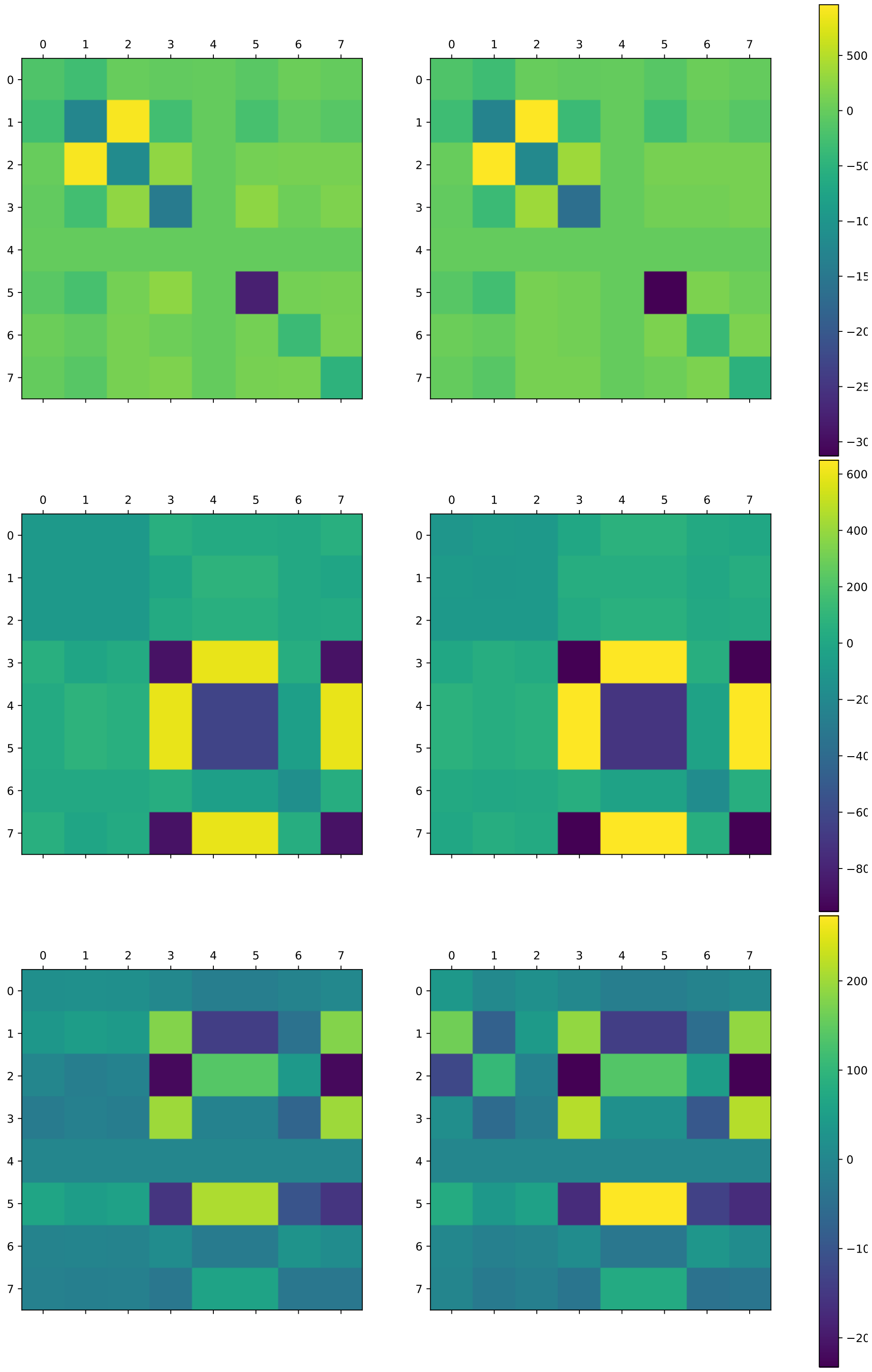

Figure 0.6: The comparison between the exact $J = C^{-1}$ and the approximated J computed as in equation 0.23. Left column: approximated J. Right column: exact J. First, second and third row: $J^{(\mathrm{xx})}$, $J^{(\mathrm{yy})}$ and $J^{(\mathrm{xy})}$ respectively.

considered, and inferred from, despite they are redundant. The resulting experimental correlation matrix C is singular as it exhibits $D - r$ null eigenvalues, each one corresponding to a constraint. However, the influence of the constraints on the inferred model is subtracted by defining a probability distribution in the subspace of the coordinates that are invariant under the linear operation associated to the constraint. Mathematically, this is done through the pseudo-inverse operation (see eq. 0.14), which discards the subspace expanded by the eigenvectors corresponding with null eigenvalue. The corresponding inferred probability distribution corresponds to a system which is

invariant under rescaling of the constraints c_j, equation (0.9).

An alternative method to infer P avoiding the influence of constraints consists in inferring only a subset of r non-redundant, unconstrained variables, in terms of which the correlation matrix has rank equal to r. As mentioned before and in the main article, this method may lead to a matrix of effective interactions leading to a less clear *interpretation.* The J_{ij} elements will reflect in this case the influence of the constraints in the considered r variables. Oppositely, with the null-mode subtraction method, the J matrix represents a system which already satisfies the constraint (see section 0.4) and, for this reason, the J_{ij} matrix elements do not reflect its influence on the data.

An illustration of these concepts is shown in the main article, where we compare matrices C and J. The null-mode subtraction method provides a matrix J which is actually sparser than matrix C. This does not occur when inferring from a reduced, non-redundant set of variables.

A further, particularly clear illustration is seen in terms of inter-landmark distances $\mathbf{d} = (d_i)_{i=0}^{10}$, an alternative parametrization of the facial vectors $\boldsymbol{\Delta}$ (see the precise definition in [8] and in figure 0.2) in terms of 11 vertical or horizontal distances separating couples of landmarks. The function that maps a vector of inter-landmark distances $\mathbf{d}$ into a vector of landmark coordinates $\boldsymbol{\Delta}$ is one-to-one (and depends on some distances of the reference portrait). The distances $\mathbf{d}$ are subject to a constraint, reflecting the scale invariance of the problem [8]: $\sum_{i=1}^{4} d_i = 1$, which signifies that all the distances d_i are in units of the total face length (see figure 0.2). This constraint induces a null mode in the correlation matrix.

We now compare the effective interaction matrices corresponding to the two alternative ways of inference discussed before. We first calculate, see figure 0.7, the matrices $J^{(-k)} = {C^{(-k)}}^{-1}$, the inverse of the $D-1 \times D-1$ correlation matrices $C_{ij}^{(-k)} = \langle d_i d_j \rangle$ in which the k-th row and column have been removed, $i,j \neq k$. The matrices $J^{(-k)}$ are presented in figure 0.7 for $k = 1,2,3,4$, compared with matrices $C^{(-k)}$.

We observe that the variables involved in the constraint result to be anticorrelated, $C_{ij}^{(-k)} < 0$ when both i,j are in the set $1,2,3,4$, a fact fact may be attributed to the presence of the constraint (e.g., vectors with larger distances d_1 tend to exhibit lower d_2's, since, for all vectors, $d_1 + d_2 + d_3 + d_4 = 1$). Indeed, also $-J_{ij}^{(-k)} < 0$ for $1 \leq i,j \leq 4$ and such that $i,j \neq k$, $i \neq j$: it is necessary an *anti-ferromagnetic interaction*, or a statistical tendency of variable i to decrease when variable j increases, in order that the theoretical distribution associated to $J^{(k)}$ describes the statistics of the set of variables. Such statistical tendency is **on the top of other statistical tendencies, of cognitive origin, not related to the constraint**. In other words, the matrices $J^{(-k)}$ describe the data statistics of two different origins: those associated to the constraint, and those of cognitive origin.

Second, in figure 0.8 we present the resulting effective interaction with the null-mode subtraction method, using as J the pseudo-inverse of matrix C. We observe that, interestingly, all the couplings $-J_{ij} > 0$ when both i,j belong to the set $\{1,2,3,4\}$, implying a *ferromagnetic effective interaction* in the physical language, or a *positive* tendency of d_i to increase when d_j increases. In the pseudo-inverse case, J represents the statistical effective interaction *with the influence of the constraint subtracted.* We learn information of *cognitive*, "physical" origin from J, that was veiled in the matrices $J^{(-k)}$'s, influenced by the presence of the constraint. In particular, we learn that the experimental subjects tend to prefer higher eyes, higher d_1, in facial modifications with larger noses, larger d_2.

In conclusion, the method of null-mode removal that we have used in this work allows, in general, for a more faithful *interpretation* of the effective parameters with respect to the alternative method of inferring from a set of non-redundant variables. It is important to stress that the generative model obtained from J and from $J^{(-k)}$ is expected to be equally faithful. In other words, the difference discussed in this section regards but the interpretation of J_{ij} elements, and only in situations in which the parameters actually admit an interpretation, as it is the case in the present and in other problems in biophysics and neurophysics. Indeed, the efficiency of both generative models as a classifier in the two groups of subjects (male/female, $\mathcal{S} = \mathcal{S}_A \cup \mathcal{S}_B$), results to be equivalent, see section 0.16.

0.9 Average proportions and pairwise correlations

Facial beauty has been related to proportions since the Renaissance [9, 10], and most modern machine learning studies pose the problem in terms of proportions too [11, 12, 13, 14, 15, 16].

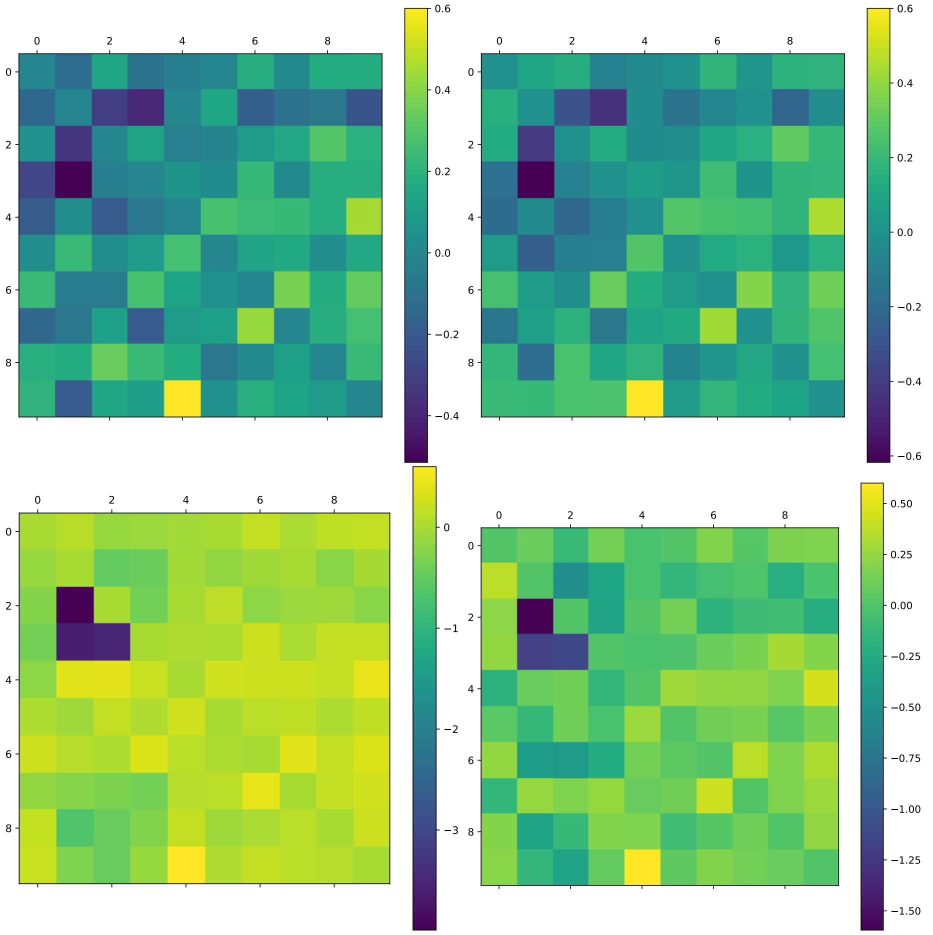

Figure 0.7: t-value corresponding to the matrices $C^{(-k)}$ (upper right triangle) and $J^{(-k)}$ (bottom left triangle), in terms of inter-landmark distances, and avoiding the k-th distance, d_k. Top left, top right, bottom left and bottom right correspond, respectively, to $k = 1, 2, 3, 4$. We can observe that, for $i, j = 1, 2, 3, 4$, all the elements of J_{ij} are positive. The diagonals of all matrices have been set to zero for a clearer comparison.

In the main text we have explained that the dataset is faithfully described by a MaxEnt probability distribution $\mathcal{L}(\mathbf{x}|J, \mathbf{h})$, whose sufficient statistics is the matrix of pairwise correlations. We have also argued that, for a complete statistical description of the database of facial modifications, a model based on pairwise correlations is not enough. This implies that *proportions*, or ratios among facial distances, contains most of the information present in the database, although there is significant information, of cognitive origin, beyond proportions. We here justify such statement, making notice that the information regarding facial proportions is codified in the matrix of correlations among couples of facial distances.

Consider two facial coordinates, r_α, r_β, referring to the x or y coordinates of two landmarks, say i and j. We will consider their ratio, r_α/r_β, which is the mathematical expression of a proportion. Calling $\bar{r}_\alpha = \langle r_\alpha \rangle$ the experimental average value, one has $r_\alpha = \bar{r}_\alpha + \Delta_\alpha$, by definition of displacement Δ_α. The displacements around the average, Δ_α, are much lower than the averages $\bar{r}_\alpha$, for all coordinate, α (see [8]). This justifies a Taylor expansion of r_α/r_β for low Δ's. Indeed, to the second order in the Δ's:

$$\frac{r_\alpha}{r_\beta} = \frac{\bar{r}_\alpha}{\bar{r}_\beta}\left(1 - \frac{\Delta_\beta}{\bar{r}_\beta}\right) + \frac{\Delta_\alpha}{\bar{r}_\beta} - \frac{\Delta_\alpha \Delta_\beta}{\bar{r}_\beta} + O\left[\left(\frac{\Delta}{r}\right)^2\right] \tag{0.28}$$

The experimental average of this expression $\langle r_\alpha/r_\beta \rangle$ is, up to an additive constant, equal to $-(1/\bar{r}_\beta^2)\langle \Delta_\alpha \Delta_\beta \rangle$ (having used that $\langle \Delta_\alpha \rangle = 0$). Hence, the average proportions are completely determined, in the case of small displacements, by the pairwise correlations.

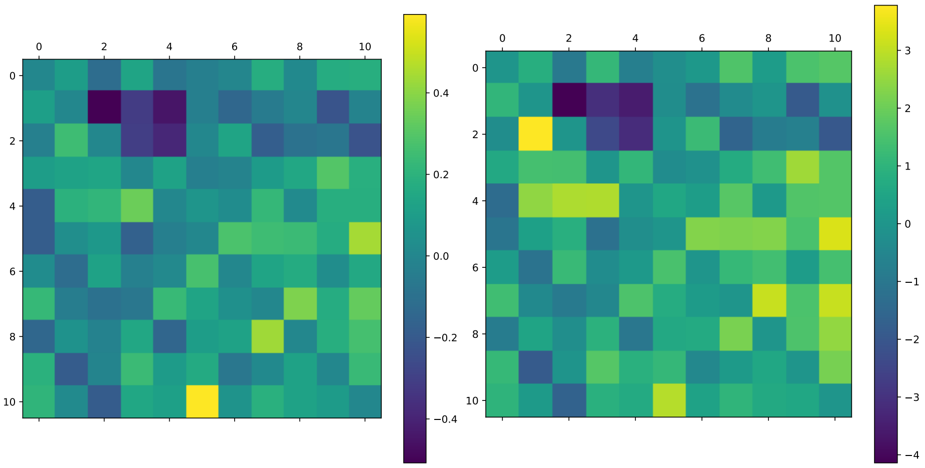

Figure 0.8: Matrices C (upper right triangle) and J (bottom left triangle) in terms of inter-landmark distances, using the whole, redundant set of distances and the null-mode subtraction method. Left: the matrix elements. Right: their t-values. We see, at opposite with figure 0.7, that $J_{ij} < 0$ for $i, j = 1, 2, 3, 4$. The diagonals have been set to zero for clearer comparison.

0.10 Harmonic interactions and elastic constants

In the main article we have explained that the 2-MaxEnt model for vectors of facial distance displacements $\boldsymbol{\Delta}$ may be interpreted as the Maxwell-Boltzmann distribution corresponding to a set of particles in 2D interacting through a set of three anisotropic, couple-dependent springs, in the canonical ensemble. We will here justify such statement. We will focus in a couple of landmarks, say i, j. We will call x_i, x_j the components of the position of landmarks i, j over two versors in the plane, $\hat{e}^{(i)}$, $\hat{e}^{(j)}$, respectively. In other words: $x_i = \vec{r}_i \cdot \hat{e}^{(i)}$ and the same for j, in the notation of the main article. Given the coordinates x_i, x_j, and if $\hat{e}^{(i)} = \hat{e}^{(j)}$, the quantity $\delta_{ij} = x_i - x_j - (\bar{x}_i - \bar{x}_j)$ is the change in the distance among i and j with respect to the average vector, and along the common axis $\hat{e}^{(i)}$. For example, if the versor is the vertical axis, δ_{ij} indicates the shift of the vertical distance among landmarks i, j with respect to the average distance among i, j. We will define *the elastic interaction energy as* $(1/2)k_{ij}\delta_{ij}^2$, which is minimum and equal to zero whenever the distance among i, j is unchanged with respect to the average, $\delta_{ij} = 0$, regardless on the single-landmark displacements x_i, x_j. We make notice that expanding, again, in $\delta_i = x_i - \bar{x}_i$ to the second order in δ_i and δ_j, it is: $\delta_{ij}^2 = -2\delta_i\delta_j + b + O[\delta^2]$, where b is a constant in δ_i and δ_j, depending only on $\bar{x}_i$ and $\bar{x}_j$. Henceforth, the elastic interaction energy, $\mathcal{E} = (1/2)k_{ij}\delta_{ij}^2$ is, up to a constant, and for small fluctuations around the average, $= -2\delta_i\delta_j k_{ij}$, which is the form of the interaction energy in the pairwise Hamiltonian model with the following relation among elastic constant and effective interaction matrix element: $k_{ij} = -(1/2)J_{ij}$. Fixing, for example, $\hat{e}^{(i)} = \hat{e}^{(j)} = \hat{e}_{ij}$, the versor joining the average position of both landmarks, $\hat{e}_{ij} = \langle \vec{r}_i - \vec{r}_j \rangle / |\langle \vec{r}_i - \vec{r}_j \rangle|$, we have that $k_{ij}^{||} = -(1/2)J_{ij}^{||}$, and idem for the perpendicular components of $\vec{r}$, $\perp$, and for the vertical, horizontal and oblique components of $\vec{r}$.

0.11 Cognitive origin of non-linear correlations

In the experiments presented in [8], the subject sculpts her/his ideal facial modification through the interaction with a software called FACEXPLORE, based on genetic and image deformation algorithms. The sculpture process consists in a sequence of multiple left/right choices among couples of facial images, eventually leading to an estimation of the ideal modification according to the subject. Actually, the genetic algorithm performs the **recombination** and **mutation** steps, while the single experimental subject actually plays the role of the **selection** step, through her/his choices.

The genetic algorithm used (called Differential Evolution) processes different coordinates independently (see the SI of [8]). The only correlation among coordinates is expected to be induced by the **selection** process, performed by the human subject. As a consequence, one should expect that the only origin of correlations among coordinates in the populations sculpted by subjects (by

the same or by different subjects) are of cognitive nature.

In fact, this is not the case: *part* of the correlations that one observes experimentally are due to an artifact of the algorithm. In a null-model experiment with a *random* sequence of left/right choices, the resulting database exhibits significant non-linear correlations of order 2 and 3 among facial coordinates. The correlations of order three, $\langle \Delta_i \Delta_j \Delta_k \rangle$, are statistically compatible with the 3-order correlations observed in the human experiment [8].

The solution of this paradox is that, while the genetic algorithm does not introduces correlations in the recombination and mutation steps, it actually may amplify the correlations among facial coordinates which are present in the initial condition of the null-model genetic population of facial vectors. Such initial populations are trivially correlated, since some constraints were imposed in the definition of the face space: mainly $\sum_{i=1}^{4} d_i = 1$ and $d_{10} < d_5$ (see section 0.8 and figure 0.2).

In reference [8] we proposed a method to "subtract" the influence of the *a priori*, non-cognitive or artifact correlations present in the null model experiment, from the cognitive true correlations that we observed. The method revealed that the artifact **pairwise** correlations did not have a significant impact in the results. We suspect that correlations of higher order may be, instead, significantly influenced by the artifact effect.

In the main article text, we have explained that non-linear inference algorithms allow for a much better classification of the database according to the gender of the experimental subject. This fact implies that, quite interestingly, the differences between facial vectors sculpted by males and by females is encoded in non-Gaussian correlations, beyond proportions ($p = 2$), beyond triplets and perhaps quadruplets of facial distances. In the main article, we have also explained that this **may imply** that such differences codified in non-Gaussian correlations are of cognitive order, i.e., that male and female subjects' do prefer facial variations differing in non-Gaussian correlations and, in particular, that humans evaluate quantities that are much more complicated than proportions, when forming an impression about a face. The fact that the introduction of non-linearity helps in a gender classification task, which reflects real and well-known cognitive differences, may suggest so.

An alternative explanation is that the distinguishable differences among male and female preferred facial variations are all codified in pairwise effective interactions only (say, roughly speaking, that males and females differ only in the J matrix, if it could be measured without bias). The non-linear interactions would turn anyway relevant for the classification, since the correlations propagated by the genetic algorithm are coupled to the ones induced by the subject: subjects differing only in J would **also** induce, by means of the artifact, differences in the correlations of higher order.

Further experiments are needed to clarify this issue.

0.12 Generality of the unsupervised inference models

Crucially, the two models of unsupervised inference presented in the main text exhibit a wide generality, going beyond the particular database that we infer in this work. (1) First, the inferred set of facial vectors may be composed by facial images selected according to any criterion: selected by a pool of subjects among real facial images or by a single individual (in this case the distribution $\mathcal{L}(\cdot|\boldsymbol{\theta})$ would probabilistically characterise the single subject's preferred region in face-space); selected according to a criterion different from attractiveness; even not having been selected by subjects but chosen according to some objective criterion as age or gender ($\mathcal{L}(\mathbf{f}|\boldsymbol{\theta})$ would hence represent the probability that a facial image characterised by the facial vector $\mathbf{f}$ presents the desired feature). (2) Second, they can be used to infer any other database of images characterised by the geometric positions of facial (or, in general body) landmarks. (3) Third, these models may be immediately extended to process also non-geometric degrees of freedom (treating the texture and geometric degrees of freedom on the same footing [17]).

0.13 Learning in the non-linear MaxEnt model.

The 3-MaxEnt model parameters are $n_{\mathrm{p}} = [D] + [D(D+1)/2] + [(D^3 - D^2)/6 + D] = D^3/6 + D^2/3 + 5D/2$ independent components of the interaction tensors $\boldsymbol{\theta} = (\mathbf{h}, J, Q)$ of order 1,2,3. Their value in the article is fixed by Maximum Likelihood, equation (0.3). In the case of the 3-MaxEnt model, we have estimated the maximum likelihood value of the parameters $\boldsymbol{\theta}^*$ by means of a numerical maximisation of the joint database likelihood by deterministic gradient ascent (see also [18]).

A discrete sequence of interaction tensors $\boldsymbol{\theta}(t)$ are recursively updated according to a deterministic gradient ascent rule: $\boldsymbol{\theta}(t+1) = \boldsymbol{\theta}(t) + \eta_{\boldsymbol{\theta}}\, \partial_{\boldsymbol{\theta}} \left[\ln \mathcal{L}(\mathcal{S}|\boldsymbol{\theta})\right]_{\boldsymbol{\theta}(t)}$, using a learning rate $\eta_{\boldsymbol{\theta}}$ depending on the tensor that is being updated. We use $\eta_J = 10^{-2}$ for the matrices and $\eta_Q = 10^{-3}$ for the 3-order tensors.

At a given epoch t of the gradient ascent iteration, the gradient of the joint likelihood with respect to the effective interaction components involves a theoretical correlator (of order 1,2 or 3) according to the current value of the couplings. For instance: $\left[\partial_{J_{\alpha\beta}} \ln \mathcal{L}(\mathcal{S}|\boldsymbol{\theta})\right]_{\boldsymbol{\theta}(t)} = \langle \Delta_\alpha \Delta_\beta \rangle_{\mathcal{L}(\cdot|\boldsymbol{\theta}(t))} - \langle \Delta_\alpha \Delta_\beta \rangle$. Such theoretical correlator is in its turn estimated by means of a Markov Chain Monte Carlo (MCMC) Metropolis algorithm for the sampling of configurations from the theoretical distribution at the corresponding epoch, $\mathcal{L}(\cdot|\boldsymbol{\theta}(t))$. For such MCMC algorithm, we use a number of sweeps $= 10^6$ in each epoch. The MCMC vectors $\boldsymbol{\Delta}$ are initialised as normal variables with variance equal to their empirical variance, and the Metropolis trials are chosen uniformly in the interval $\Delta_\alpha \in [-6\sigma_\alpha, 6\sigma_\alpha]$, where σ_α is the empirical standard deviation of Δ_α. This corresponds to maximising the likelihood function:

$$\mathcal{L}(\boldsymbol{\Delta}|\boldsymbol{\theta}) = \frac{1}{Z} e^{-H(\boldsymbol{\Delta}|\boldsymbol{\theta})} \mathsf{H}(\boldsymbol{\Delta}|\mathbf{B}) \tag{0.29}$$

where H is the multivariate Heaviside function in the hyper-parallelepiped centered in the origin $\boldsymbol{\Delta} = \mathbf{0}$ and of side lengths $2B_\mu = 12\sigma_\mu$, and where $H(\cdot|\boldsymbol{\theta}) = H_2(\cdot|\boldsymbol{\theta}_2) + H_3(\cdot|Q)$. Finally, in order to monitor the state the convergence to the stationary state, in every step of the gradient ascent maximisation we evaluate the difference of the empirical average of the Hamiltonian and its expected value according to $\mathcal{L}(\cdot|\boldsymbol{\theta})$,

$$\Delta H(t) = \langle H(\cdot|\boldsymbol{\theta}(t)) \rangle - \langle H(\cdot|\boldsymbol{\theta}(t)) \rangle_{\mathcal{L}(\cdot|\boldsymbol{\theta}(t))} \tag{0.30}$$

the iteration stops when $|\Delta H(t)|$ decreases below a certain threshold [19, 18]. The condition $\Delta H = 0$ is necessary for the maximum likelihood condition. As initial values of the learning dynamics, we choose $\mathbf{h} = \mathbf{0}$, $J = \mathbb{I}_D$, $Q = 0$.

The value of $B_\mu = 6\sigma_\mu$ has been chosen so that all the empirical vectors lie in the parallelepiped $\mathbf{B}$ and, at the same time, the maximum likelihood probability distribution exhibits a single local maximum in its support $\mathbf{B}$. Indeed, the probability distribution projected along the data principal components (Δ'_μ or the projection of $\boldsymbol{\Delta}$ on the μ-th eigenvector of J), is qualitatively a perturbed normal distribution with variance σ_μ^2 (obtained for $Q = 0$), with asymmetric and fatter tails (see [18]).

0.14 Learning the database with the Gaussian Restricted Boltzmann Machine

Definition of the model. The Gaussian Restricted Boltzmann Machine (GRBM) is a type of generative stochastic two-layered Artificial Neural Network [20, 21, 22, 23]. It is a generalisation of the Restricted Boltzmann Machine (RBM) model [24], that learns a probabilistic generative model for real-valued vectors: the visible neurons in the input layer, $\boldsymbol{v}$, assume real values. The value of the hidden neurons $\boldsymbol{h}$ is, instead, binary, $h_j = 0, 1$. The state of the $N_{\rm v}$ visible $\boldsymbol{v} = (v_i)_{i=1}^{N_{\rm v}}$ and $N_{\rm h}$ hidden $\boldsymbol{h} = (h_i)_{i=1}^{N_{\rm h}}$ neurons is described by an energy-based probability density:

$$p(\boldsymbol{v}, \boldsymbol{h}|\boldsymbol{\theta}) = \frac{1}{Z_{\boldsymbol{\theta}}} e^{-E(\boldsymbol{v},\boldsymbol{h}|\boldsymbol{\theta})} \tag{0.31}$$

in terms of the parameters $\boldsymbol{\theta} = \{W, \boldsymbol{b}, \boldsymbol{c}, \boldsymbol{\sigma}\}$, to be inferred in the learning process. W is a real $N_{\rm v} \times N_{\rm h}$ matrix coupling real and visible variables, while $\boldsymbol{c}$, $\boldsymbol{\sigma}$ are $N_{\rm v}$-dimensional real vectors representing the bias over the visible neurons and their standard deviation, respectively, while $\boldsymbol{b}$ is a real $N_{\rm h}$-dimensional vector representing the bias over hidden neurons. $Z_{\boldsymbol{\theta}}$ is a normalising constant, depending on the parameters. The function energy E is defined so that the conditional probability distribution $p(\boldsymbol{v}|\boldsymbol{h}, \boldsymbol{\theta})$ results to be a normal, independent distribution over visible variables. It assumes the form:

$$E(\boldsymbol{v}, \boldsymbol{h}) = -\sum_{i=1}^{N_{\rm v}} \sum_{a=1}^{N_{\rm h}} \frac{W_{ia} v_i h_a}{\sigma_i^2} + \sum_{i=1}^{N_{\rm v}} \frac{(v_i - c_i)^2}{2\sigma_i^2} - \sum_{a=1}^{N_{\rm h}} h_a b_a \tag{0.32}$$

The GRBM probabilistic generative model is obtained through a marginalisation of the hidden variables: $p(\boldsymbol{v}|\boldsymbol{\theta}) = \sum_{\boldsymbol{h}} p(\boldsymbol{v}, \boldsymbol{h}|\boldsymbol{\theta})$. This model (as far as the hidden neurons are binary) is known to induce non-linear interactions among the visible variables, up to order $p = N_{\rm v}$ in the most general case [25].

Learning protocol. We have trained the model over a set of redundant or non-redundant data, obtaining equivalent results. As a learning algorithm we have used gradient ascent through persistent contrastive divergence with $\mathsf{k} = 1$ Monte Carlo step, along with mini-batch learning with batch size B [21], and an epoch-depending variable learning rate, η, increasing linearly with the number of epochs. We have set the value of the learning hyperparameters to: number of steps $n_s = 2 \cdot 10^5$, batch size $B = 200$, momentum $\mu = 0$, initial learning rate $\eta_0 = 2 \cdot 10^{-3}$. The learning rate slope is set such that $\eta_{n_s} = 2 \cdot 10^{-5}$. The parameters W, $\boldsymbol{b}$ and $\boldsymbol{c}$ are initialized following a standard procedure [26, 27]:

$$W_{ia} = \chi_{ia} \sqrt{\frac{6}{N_V + N_{\rm h}}} \ , \quad \chi_{ia} \in (-1, 1) \quad \forall i, a \tag{0.33a}$$

$$b_a = -\frac{1}{2}(||\boldsymbol{W}_{*,a} + \boldsymbol{c}|| + ||\boldsymbol{c}||) + \log(0.1) \quad \forall a \tag{0.33b}$$

$$c_i = 0 \quad \forall i \tag{0.33c}$$

$$\sigma_i = 1/2 \quad \forall i \tag{0.33d}$$

As equilibration test we have verified that the test-set joint likelihood is stationary as a function of the number of epochs, within its associated standard deviation. We have performed an assessment of the algorithm efficiency as a function of the number of hidden neurons, $N_{\rm h}$. As shown in figure 0.9, both the test and training-set joint likelihood exhibit a monotonous increasing behaviour vs $N_{\rm h}$, showing no sign of severe overfitting. The auROC score saturates at its maximum value for values $N_{\rm h} \gtrsim 8N_{\rm v}$, with $N_{\rm v} = 10$, confirming this picture. We have consequently considered, for the analysis performed in the main article, $N_{\rm h} = 100$.

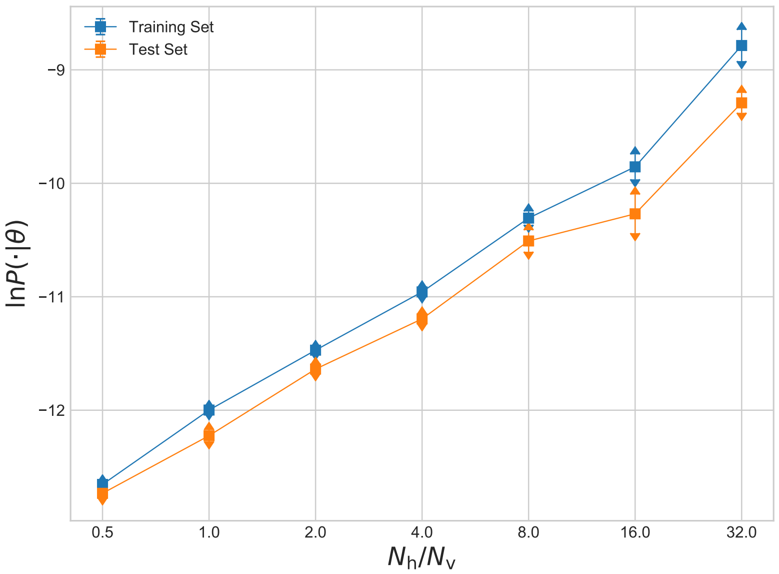

Figure 0.9: Training and test-set log-likelihood as a function of the ratio $N_{\rm h}/N_{\rm v}$, each point has been obtained averaging over 5 realizations of the learning, once the stationary state of the train log-likelihood has been achieved.

Before GRBM learning, the data has been pre-processed eliminating 6 redundant coordinates, subtracting the average (of the whole database, not of the $\{A, B\} \times \{\text{train}, \text{test}\}$ datasets separately) and standardising, or dividing each vector component-wise by the vector of standard deviations

Table 0.1:

algorithm	auROC
random forest	0.995
GRBM	0.988
3-MaxEnt	0.930
2-MaxEnt	0.848
2-MaxEnt non-redundant	0.846
2-MaxEnt approximated	0.830
2-MaxEnt null-xy	0.770
2-MaxEnt dot	0.745
1-MaxEnt	0.654

along the whole set. We have learned the dataset with a variable number of hidden neurons N_{h}, from $N_{\mathrm{h}} = D$ to $N_{\mathrm{h}} = 16\,D$.

Afterwards, for the sake of the classification, the model has been trained over the A, B training databases separately, leading to two sets of parameters $\boldsymbol{\theta}_A$, $\boldsymbol{\theta}_B$ and, consequently, to a likelihood function $\mathcal{L}_{\mathrm{RBM}}(\cdot|\boldsymbol{\theta}_{A,B})$. Afterwords, the score $\mathsf{s}(\tilde{\mathbf{r}}) = \ln \mathcal{L}(\tilde{\mathbf{r}}|\boldsymbol{\theta}_A) - \ln \mathcal{L}(\tilde{\mathbf{r}}|\boldsymbol{\theta}_B)$ is defined for every standardised and non-redundant vector $\tilde{\mathbf{r}}$ of the A, B test-sets. Such score is used to construct the ROC curve and scores shown in the main article.

0.15 Classification with the Random Forest algorithm

In the random forest classification presented in the main article and in figure 0.10, we have used the Random Forest Classifier [28, 29], using 1000 trees created from bootstrapped sub-sample and with nodes expanded until all leaves are pure. As an assessment of the single-split quality we have considered the Gini function. The number of random features considered in the best split choice is equal to $\mathsf{int}(\mathsf{sqrt}(D))$, where $D = 16$ is the number of features.

0.16 Detailed comparison among several classification methods

We now present a more detailed analysis of all the classification algorithms that we have considered for the classification of the database according to the subjects' gender. In table 0.1 we present a systematic comparison of the auROC value [30], a standard estimator of the classification accuracy (the area under the corresponding ROC curves in figure 0.10), associated to the classification according the various algorithms. In particular, *2-MaxEnt approximated* is the 2-Maxent model resulting from the approximation in equation (0.23); *2-MaxEnt null-*xy is the model consisting neglecting the oblique interactions, $J^{(\mathsf{xy})} = 0$; *2-MaxEnt dot* is defined in equation (0.27); *1-MaxEnt dot* is defined by inferring the external fields only (and taking the interaction matrix J, required for the normalisation of P, as a diagonal matrix whose diagonal is equal to the inverse variance of each variable).

The results of table 0.1 and of figure 0.10 confirm the picture presented in the main article. The value of the single facial distances (in units of the facial length) are not enough for an accurate description of the database of facial modifications. The introduction of pairwise effective interactions, which explain proportions, or ratios of facial coordinates, induces a notable improvement in the statistical description. Moreover, *oblique* effective interactions (coupling the x coordinate of one landmark with the y coordinate of another landmark) result a fundamental ingredient. Finally, a crucial role, at least for the sake of the classification according to the subjects' gender, is plaid by effective interactions of higher order: $p = 3$ (3-MaxEnt) and $p > 3$ (GRBM and random forest). We conclude that the classification is a valid method for the assessment of the assessment of the relative relevance of the various terms.

Remarkably, an as we anticipated in section 0.8, the algorithm used to avoid the constraints (inferring from a reduced, non-redundant set of variables, or using the null-mode subtraction method) do not change the efficiency of the classification. Indeed, the model that we call *2-MaxEnt non-redundant* in table 0.1 and in figure 0.10 is identical to *2-MaxEnt* but in terms of a subset of 10 non-redundant variables. Its auROC estimator and ROC curve are statistically distinguishable from *2-MaxEnt* (with 16 variables and null-mode subtraction).

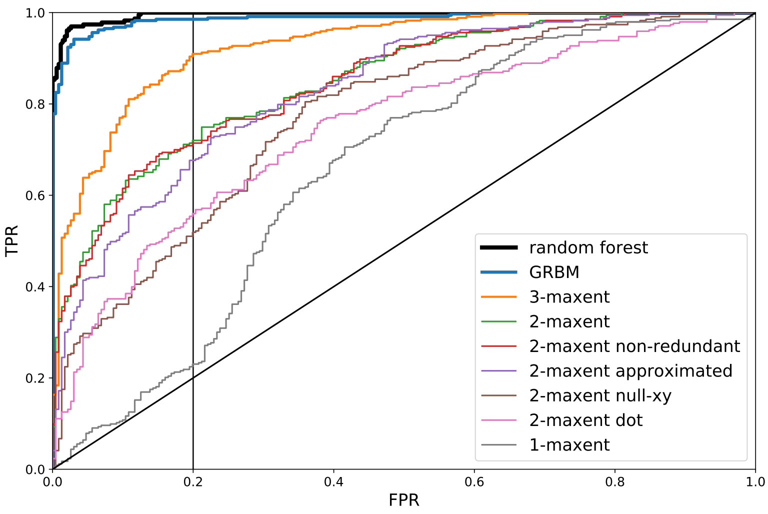

Figure 0.10: ROC curves of all the models defined in the text. The order of the model in the legend (and in table 0.1) is also the order with which the corresponding curve crosses the vertical line at FPR=0.2.

0.17 Inter- and intra-subject correlations and errors.

The set of facial vectors sculpted by a single subject, $\{\mathbf{r}^{(v,i)}\}_{i=1}^{\mathcal{N}}$, are not the result of independent sculpting experiments. They are, rather, correlated as far as they are the outcome genetic *population* of facial vectors that evolved according to a stochastic evolutionary algorithm coupled to a sequence of choices performed by the experimental subject [8]. Consequently, it is crucial to subtract the effect of intra-subject (or intra-genetic population) correlations among facial vector components from the inter-subject correlations. On the one hand, one may define the *bare correlation* matrix, accounting from both sources of correlations, defined by summing over both subject and population indices: $C_{\alpha\beta} = \langle \Delta_\alpha \Delta_\beta \rangle$. On the other hand, the *inter-subject correlation matrix* accounts only for the inter-subject correlation, and is defined as $\bar{C}_{\alpha\beta} = \overline{(1/n_{\rm s}) \sum_{v'=1}^{n_{\rm s}} \Delta_\alpha^{v(v'),i(v')} \Delta_\beta^{v(v'),i(v')}}$, where $v(v')$ and $i(v')$ are random indices in the sets $1,\dots,n_{\rm s}$ and $1,\dots,\mathcal{N}$ respectively, uncorrelated among them and on v', and the overline $\bar{\cdot}$ means an average over a sufficiently high number of realisations of the set of indices $v(v')$, $i(v')$ for $v' = 1,\dots,n_{\rm s}$. The statistical uncertainty associated to the inter-subject correlation, $\sigma_{C_{\alpha\beta}}$, is the standard deviation of the overline argument under many realisations of the set of indices (in other words, a Bootstrap error using only one vector for subject in each Bootstrap sampling, see the SI of ref. [8]). Consequently, the error associated to the inter-subject correlation is of order $\sim {n_{\rm s}}^{-1/2}$, and not of order $\sim S^{-1/2}$ as that of the bare correlation matrix. Analogously, we also define inter-subject and bare 3-component correlations.

If the inferred model should describe the probability of a given facial vector to have been selected by any subject in the database, then it should be committed to reproduce by construction the inter-subject (not the *bare*) correlations. Otherwise, the probabilistic generative models may also simply describe the whole set of facial vectors in the [8] experiments, hence accounting also for the intra-subject correlations; the corresponding MaxEnt models would reproduce by construction the 2 or 3 bare correlations in this case. In our data analysis software one can specify whether the $2,3$ MaxEnt inferred model reproduce *bare* or *inter-subject* correlations. In this article, some results (the reproduction of angle histograms and the analysis of J matrices) correspond to the inter-subject inference models. The classification tests have, instead, been done with the bare models. For the sake of classification, we have simply tested the ability of the algorithm to capture any useful correlation, regardless of its origin, cognitive or algorithmic. The bare inference models suffer less from the curse of dimensionality since the effective database size is $S = \mathcal{N} n_{\rm s}$ instead of $n_{\rm s}$.

0.18 Description of the E1 experiment

We now recall a description of the experiment E1 presented in [8].

The face-space. The face-space used in [8] is based on a separation of texture/geometric degrees of freedom [14]. The face-space **facial vectors** codify the geometric degrees of freedom only (corresponding to the Cartesian coordinates $\mathbf{r}$ of the set of landmarks), while the texture degrees of freedom correspond to a real portrait called **reference portrait**. Given a vector $\mathbf{r}$ and the reference portrait, we construct, throught image deformation algorithms (based on affine linear transformations) a novel image whose landmarks occupy the positions given by $\mathbf{r}$, and whose texture is coherently and realistically deformed from that of the reference portrait. The reference portrait used in the E1 experiment, called RP1, is fixed for all the subjects.

The aim of the experiment is to provide a population of $\mathcal{N}$ facial vectors for each experimental subject. Such a population aims to be an empirical sample of the subject's preferred modifications of the reference portrait. This means that the subject probabilistically prefers facial images associated with vectors that are close to the vectors in the population, rather than local fluctuations away from it. The subject does not sculpt the population by successive discrimination among faces differing by a single coordinate, which turns out to be an inefficient strategy of face-space exploration, but rather through the interaction with a genetic algorithm, implemented by our software FACEXPLORE. In such (differential evolution) genetic algorithm, the individuals are the $\mathcal{N}$ facial vectors, their genetic code is the corresponding vector of geometric coordinates, and the selection process is performed by the subject, who iteratively select, according to her/his personal criterion, which facial vectors will survive in the next generation.

General description. The subjects sculpt their preferred variation of a facial vector, which codifies geometric coordinates. Starting from $\mathcal{N}$ initial random facial vectors, the FACEXPLORE software generates pairs of facial images (composed by each original facial vector and by a potential offspring generated by mutation and recombination). Each pair is presented to the subject, who selects the one that she/he finds more attractive. Based on $\mathcal{N}$ left/right choices, the genetic algorithm produces a successive generation of $\mathcal{N}$ vectors. This process is repeated T times, in a constant feedback loop of offspring generation and selection operated by the subject. Such iterations leads to a sequence of T generations of facial vectors, each one more adapted than the last to the subject's selection criteria, eventually converging to a pseudo-stationary regime in which the populations are similar to themselves and among consecutive generations. The T-th generation is taken as the empirical sample of the subject's preferred modifications of the reference portrait.

Some details of the experimental setup. The experiment was performed by a pool of $n_s = 95$ volunteers (54 female, 39 male, of age average and standard deviation: 26(12)), mainly students, researchers and professors of the *Sapienza* University, in Rome. Each subject performed a number of $\mathcal{N}T = 280$ choices among couples of facial images. These are uncompressed 400×300 pixel, B/W images of 72 pix/inch resolution in an 1024×768 monitor. The viewing distance is 65(10)cm. The reference portrait RP1 has been taken from the Chicago face database [31]. The experiment lasted roughly 25 minutes on average.

1 Bibliography

[1] E. T. Jaynes. "Information Theory and Statistical Mechanics". In: *Phys. Rev.* 106 (4 May 1957), pp. 620–630. DOI: 10.1103/PhysRev.106.620. URL: https://link.aps.org/doi/10.1103/PhysRev.106.620.

[2] Johannes Berg. "Statistical mechanics of the inverse Ising problem and the optimal objective function". In: *Journal of Statistical Mechanics: Theory and Experiment* 2017.8 (2017), p. 083402. URL: http://stacks.iop.org/1742-5468/2017/i=8/a=083402.

[3] H. Chau Nguyen, Riccardo Zecchina, and Johannes Berg. "Inverse statistical problems: from the inverse Ising problem to data science". In: *Advances in Physics* 66.3 (2017), pp. 197–261. DOI: 10.1080/00018732.2017.1341604. URL: https://doi.org/10.1080/00018732.2017.1341604.

[4] Andrea De Martino and Daniele De Martino. "An introduction to the maximum entropy approach and its application to inference problems in biology". In: *Heliyon* 4.4 (2018), e00596. ISSN: 2405-8440. DOI: https://doi.org/10.1016/j.heliyon.2018.e00596. URL: http://www.sciencedirect.com/science/article/pii/S2405844018301695.

[5] Elad Schneidman et al. "Weak pairwise correlations imply strongly correlated network states in a neural population". In: *Nature* 440.7087 (2006), p. 1007.

[6] Andrea Cavagna et al. "Short-range interactions versus long-range correlations in bird flocks". In: *Phys. Rev. E* 92 (1 July 2015), p. 012705. DOI: 10.1103/PhysRevE.92.012705. URL: https://link.aps.org/doi/10.1103/PhysRevE.92.012705.

[7] Leo P Kadanoff. *Statistical physics: statics, dynamics and renormalization.* World Scientific Publishing Company, 2000.

[8] M. Ibáñez-Berganza, A. Amico, and V. Loreto. "Subjectivity and complexity of facial attractiveness". In: *Scientific Reports* 9 (2019), p. 8364. URL: https://www.nature.com/articles/s41598-019-44655-9.

[9] Judith H Langlois et al. "Maxims or myths of beauty? A meta-analytic and theoretical review." In: *Psychological bulletin* 126.3 (2000), p. 390.

[10] Farhad B. Naini, James P. Moss, and Daljit S. Gill. "The enigma of facial beauty: Esthetics, proportions, deformity, and controversy". In: *American Journal of Orthodontics and Dentofacial Orthopedics* 130.3 (Sept. 1, 2006), pp. 277–282. ISSN: 0889-5406. DOI: 10.1016/j.ajodo.2005.09.027. URL: http://www.sciencedirect.com/science/article/pii/S0889540606006263 (visited on 10/05/2017).

[11] Hatice Gunes and Massimo Piccardi. "Assessing facial beauty through proportion analysis by image processing and supervised learning". In: *Science Direct* 7 (2006). DOI: http://dx.doi.org/10.1016/j.ijhcs.2006.07.004.

[12] Jintu Fan et al. "Prediction of facial attractiveness from facial proportions". In: *Pattern Recognition* 45.6 (2012). Brain Decoding, pp. 2326–2334. ISSN: 0031-3203. DOI: http://dx.doi.org/10.1016/j.patcog.2011.11.024. URL: http://www.sciencedirect.com/science/article/pii/S003132031100478X.

[13] F. Chen and D. Zhang. "Evaluation of the Putative Ratio Rules for Facial Beauty Indexing". In: *2014 International Conference on Medical Biometrics.* 2014 International Conference on Medical Biometrics. May 2014, pp. 181–188. DOI: 10.1109/ICMB.2014.38.

[14] A. Laurentini and A. Bottino. "Computer analysis of face beauty: A survey". In: *Computer Vision and Image Understanding* 125 (2014), pp. 184–199. DOI: 10.1016/j.cviu.2014.04.006.

[15] Hui Shen, Desmond K.P. Chau, and Jianpo et all. Su. "Brain responses to facial attractiveness induced by facial proportions: evidence from an fMRI study". In: *Nature* 6 (2016). DOI: http://doi.org/doi:10.1038/srep35905.

[16] Hui Shen et al. "Brain responses to facial attractiveness induced by facial proportions: evidence from an fMRI study". In: *Scientific Reports* 6.1 (Dec. 2016). ISSN: 2045-2322. DOI: 10.1038/srep35905. URL: http://www.nature.com/articles/srep35905 (visited on 09/29/2017).

[17] Le Chang and Doris Y. Tsao. "The Code for Facial Identity in the Primate Brain". In: *Cell* 169.6 (June 1, 2017), 1013–1028.e14. ISSN: 0092-8674, 1097-4172. DOI: 10.1016/j.cell.2017.05.011. URL: http://www.cell.com/cell/abstract/S0092-8674(17)30538-X (visited on 10/03/2017).

[18] Bernardo Monechi, Miguel Ibáñez-Berganza, and Vittorio Loreto. "Hamiltonian Modeling of Macro-Economic Urban Dynamics". In: *Royal Society Open Science* (2020, in press). arXiv: 2001.05725 [physics.soc-ph].

[19] Yann LeCun et al. "A tutorial on energy-based learning". In: *Predicting structured data* 1.0 (2006).

[20] Paul Smolensky. "Information processing in dynamical systems: Foundations of harmony theory". In: *Parallel Distributed Process* 1 (Jan. 1986).

[21] Asja Fischer and Christian Igel. "An Introduction to Restricted Boltzmann Machines". In: Jan. 2012, pp. 14–36. DOI: 10.1007/978-3-642-33275-3_2.

[22] David H. Ackley, Geoffrey E. Hinton, and Terrence J. Sejnowski. "A learning algorithm for boltzmann machines". In: *Cognitive Science* 9.1 (1985), pp. 147–169. ISSN: 0364-0213. DOI: https://doi.org/10.1016/S0364-0213(85)80012-4. URL: http://www.sciencedirect.com/science/article/pii/S0364021385800124%22.

[23] Geoffrey Hinton. "A practical guide to training Restricted Boltzmann Machines". In: (2010). URL: https://www.cs.toronto.edu/~hinton/absps/guideTR.pdf.

[24] Pankaj Mehta et al. "A high-bias, low-variance introduction to Machine Learning for physicists". In: *Physics Reports* 810 (2019). A high-bias, low-variance introduction to Machine Learning for physicists, pp. 1–124. ISSN: 0370-1573. DOI: https://doi.org/10.1016/j.physrep.2019.03.001. URL: http://www.sciencedirect.com/science/article/pii/S0370157319300766.

[25] Guido Cossu et al. "Machine learning determination of dynamical parameters: The Ising model case". In: *Phys. Rev. B* 100 (6 Aug. 2019), p. 064304. DOI: 10.1103/PhysRevB.100.064304. URL: https://link.aps.org/doi/10.1103/PhysRevB.100.064304.

[26] Nan Wang, Jan Melchior, and Laurenz Wiskott. "Gaussian-binary Restricted Boltzmann Machines on Modeling Natural Image Statistics". In: *CoRR* abs/1401.5900 (2014). arXiv: 1401.5900. URL: http://arxiv.org/abs/1401.5900.

[27] Jan Melchior. "Learning Natural Image Statistics with Gaussian-Binary Restricted Boltzmann Machines". MA thesis. Ruhr-Universitat Bochum, 2012. URL: https://www.ini.rub.de/PEOPLE/wiskott/Reprints/Melchior-2012-MasterThesis-RBMs.pdf.

[28] L. Breiman. "Random Forests". In: *Machine Learning* 45 (2001), pp. 5–32.

[29] F. Pedregosa et al. "Scikit-learn: Machine Learning in Python". In: *Journal of Machine Learning Research* 12 (2011), pp. 2825–2830.

[30] Kevin Murphy. *Machine Learning: A Probabilistic Perspective.* The MIT Press, 2012.

[31] Debbie S. Ma, Joshua Correll, and Bernd Wittenbrink. "The Chicago face database: A free stimulus set of faces and norming data". en. In: *Behavior Research Methods* 47.4 (Dec. 2015), pp. 1122–1135. ISSN: 1554-3528. DOI: 10.3758/s13428-014-0532-5. URL: https://link.springer.com/article/10.3758/s13428-014-0532-5 (visited on 05/16/2018).
